# The Treatment of Indirect Carotid Cavernous Fistula in a Pediatric Patient With Deep Recurrent Ophthalmic Artery and Hemophilia

**DOI:** 10.7759/cureus.73803

**Published:** 2024-11-16

**Authors:** Oscar A Panameño, Jesus Morales

**Affiliations:** 1 Neurological Surgery, Hospital Jose Eleuterio Gonzalez, Universidad de Nuevo Leon, Monterrey, MEX

**Keywords:** anatomical variant, carotid-cavernous fistulas (ccfs), endovascular procedure, flow-diverter stent, hemophilia-a

## Abstract

The treatment of indirect carotid-cavernous fistula (CCF) poses a unique challenge. Currently, endovascular interventions remain the principal treatment option with high cure rates and acceptable safety profiles. The anatomical characteristics of individual cases determine the optimal vascular access routes (transvenous vs. transarterial), and the devices utilized to achieve therapeutic goals. We present a rare case of an indirect CCF in a 13-year-old patient with an anatomical variant of the ophthalmic artery and hemophilia. CCFs are infrequent in the pediatric population, and an association with a deep recurrent ophthalmic artery (DROA) and hemophilia presents a unique treatment challenge.

The patient had been diagnosed with type A hemophilia in 2013. In November 2022, he had presented with insidious conjunctival hyperemia and developed gradual ocular proptosis. He had been referred to Ophthalmology in October 2023, with mild symptoms and no ocular bruit or visual acuity deterioration. Angio CT, angio MRI, and cerebral angiography at that time had shown an indirect CCF. The association of an anatomical variant with the indirect CCF led to adverse anatomy and failed transvenous/transarterial vascular access. Classic treatment options (coils, detachable balloons, and liquid embolic agents) are not feasible when selective microcatheterism is unsuccessful. A FRED flow diverter was placed in the cavernous segment of the internal carotid artery (ICA) as an off-label solution to adverse patient vascular anatomy. Successful device implantation resulted in the gradual resolution of the patient's ocular symptoms.

## Introduction

Carotid cavernous fistula (CCF) is a particular type of shunt within the cavernous sinus [1] and can be classified based on its etiology (traumatic or spontaneous), hemodynamic properties (high- or low-flow), and anatomy (direct or indirect). Barrow's classification recognizes four types based on arterial supply: type A involves a direct shunt between the cavernous segment of the internal carotid artery (ICA) and the cavernous sinus; type B is a dural shunt (indirect) between the meningeal branches of the intracavernous ICA and the cavernous sinus; type C is a dural shunt (indirect) between meningeal branches of the external carotid artery (ECA) and the cavernous sinus; and type D involves a dural shunt (indirect) between both meningeal branches of the intracavernous ICA (type B) and the meningeal branches of the ECA (type C) and the cavernous sinus [2,3]. Indirect spontaneous CCFs are less common than traumatic CCFs and even more rare in the pediatric population [4,5]. It is important to consider this etiology in patients with ocular proptosis, dilated episcleral veins, conjunctival hyperemia, chemosis, and persistent headache. While indirect CCFs usually have a benign course in the pediatric population, they still might require treatment [6,7]. The risk factors associated with spontaneous CCFs are fibromuscular dysplasia, Ehlers-Danlos syndrome type 4, and dissection of the ICA.

The classic treatment for indirect CCF includes the placement of detachable balloons, coils, and liquid embolic agents in the cavernous sinus. To achieve obliteration of the abnormal communication between dural branches and the sinus, adequate super-selective microcatheterism is necessary. We discuss a case of a patient with an anatomical variant described as deep recurrent ophthalmic artery (DROA), a rare finding with a prevalence of 0.42% in the general population [8,9], which made vascular arterial access unattainable. An alternative venous route was attempted via facial vein/superior ophthalmic vein and jugular vein/inferior petrosal, which was unsuccessful. Unachievable selective microcatheterism of the cavernous sinus prompted the implementation of a less conventional treatment approach. Flow diversion was adopted as an off-label option. This therapeutic method had to be considered with extreme caution as it requires antiplatelet therapy in a pediatric patient with hemophilia type A [10]. Reports of successful outcomes in complex cases can help neurointerventionists develop out-of-the-box strategies for adequate patient treatment.

## Case presentation

**Figure 1:**
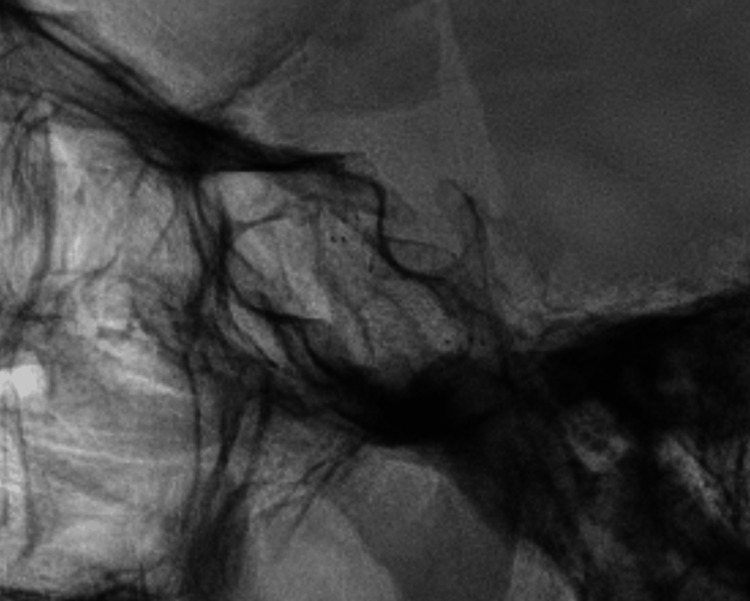
Cerebral angiography A FRED flow diverter was placed in the cavernous segment of the internal carotid artery; complete coverage of the carotid-cavernous indirect fistula

The treatment of indirect CCF is usually conservative. However, in our patient, despite an insidious clinical evolution, the absence of symptomatic resolution and worsening gradual ocular proptosis constituted indications for endovascular treatment. The specific treatment objective was the obliteration of the abnormal arteriovenous shunt at the cavernous sinus. An arterial approach was attempted through super-selective ophthalmic artery canalization. The dural branches from the deep recurrent ophthalmic artery could not be accessed because of the small caliber and elevated tortuosity. An alternative transvenous route was attempted. A facial vein/trans ophthalmic approach, and direct superior ophthalmic vein puncture were attempted. The inferior/superior petrous sinus route was unsuccessful. The FRED flow diverter involves a treatment option in Barrow type B fistulas and is safe for patients with hemophilia. A 6 Fr sheath was placed in the femoral artery and posteriorly a 6 Fr Neuron Max was placed in the left internal carotid. A Sofia intermediate catheter was utilized, and a FRED flow diverter was successfully placed through a Headway 27 microcatheter (Figure 1).

**Figure 2:**
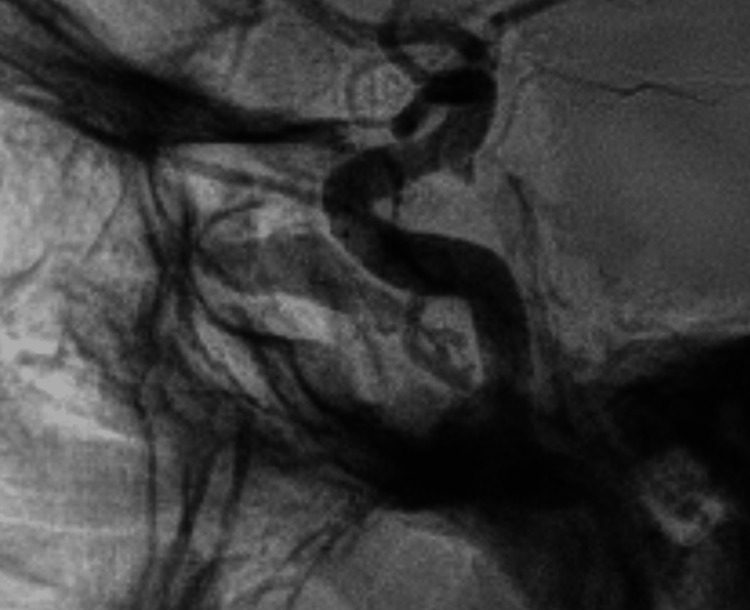
Cerebral angiography

Nonsubstraction angiography was performed with a FRED flow diverter placed in the cavernous segment of the left ICA (Figure 2).

The patient was discharged three days later without any complications. At the six-month clinical follow-up, the patient showed improvement in ocular proptosis and conjunctival hyperemia. No adverse effects were reported after the placement of the flow diverter.

## Discussion

Indirect CCFs constitute a complex vascular pathology. Its diagnosis can be challenging, particularly in the pediatric population. The development of ocular proptosis, conjunctival hyperemia, chemosis, and audible ocular bruit can be particularly insidious in this patient population. While typically these symptoms are unilateral, bilateral cases have also been reported [11].

Endovascular treatment remains the mainstay option for the treatment of indirect CCFs [12-13]. However, analyzing individual patient characteristics is necessary to determine the optimal vascular route [14] (transarterial vs. transvenous) and the embolic material to be utilized (coils, detachable balloons, and liquid embolic agents). The treatment goal is to interrupt the anomalous arteriovenous connection with the cavernous sinus. Traditional treatment options have achieved this objective by accessing the cavernous sinus and deploying embolic material [15]. However, selective microvascular access to the sinus is not always possible, especially with rare anatomical variants. In such cases, the use of flow diversion is a promising alternative in the management of specific types of CCFs. Flow diverters have been utilized in direct CCFs (Barrow type A) and indirect CCFs (Barrow type B) [16]. The treatment objective is flow redirection promoting thrombogenesis of the aberrant arterial supply to the cavernous sinus.

Our pediatric patient had an anomalous origin of the ophthalmic artery, emerging from the cavernous sinus, specifically the Inferolateral trunk. The small caliber abnormal dural branches emerged from the anatomical variant, rendering selective microcatheterization of the sinus via arterial approach impossible. Venous approach via the superior ophthalmic vein and petrous sinuses was also unsuccessful due to adverse anatomy and the presence of venous trabeculae. Flow diverter placement constitutes an alternative treatment option in such cases. After a discussion with our multidisciplinary team, the use of antiplatelet therapy, which is necessary before the placement of the flow diverter, was deemed safe in a hemophilic patient.

## Conclusions

The presence of an indirect CCF with a dural supply from a DROA in a pediatric hemophilic patient is an extremely rare occurrence. Anatomical variations pose a distinct challenge in such cases, often necessitating alternative treatment options.
